# Cancer incidence in type 2 diabetes patients - first results from a feasibility study of the D2C cohort

**DOI:** 10.1186/1758-5996-3-15

**Published:** 2011-07-13

**Authors:** Hans-Werner Hense, Hiltraud Kajüter, Jürgen Wellmann, Wolf U Batzler

**Affiliations:** 1 Institute of Epidemiology and Social Medicine; Westphalian Wilhelms University, Domagk Str. 3, 48129 Münster, Germany; 2 Epidemiological Cancer Registry for the state of North Rhine-Westphalia, Robert-Koch Str. 40, 48149 Münster, Germany

## Abstract

**Background:**

A large prospective study in patients with type 2 diabetes (T2D), the German D2C cohort, is presently being enumerated to investigate risk factors of incident cancer in diabetic patients.

**Study setting:**

A disease management program was offered, on a voluntary basis, to all T2D patients who were members of a statutory health insurance fund in Germany. This first feasibility report uses data from 26.742 T2D patients, who were 40 to 79 years old, resided in the Muenster District, and who were enrolled between June 2003 and July 2008. Cancer cases were identified through the regional Cancer Registry.

**Methods:**

Invasive cancer cases were identified using probabilistic record linkage procedures and pseudonymised personal identifiers. Censoring date was December 31, 2008. We included only first cancers, leaving 12.650 male and 14.092 female T2D with a total of 88.778 person-years (py). We computed standardised incidence ratios (SIR) for external comparisons and we employed Cox regression models and hazard ratios (HR) within the cohort.

**Results:**

We identified 759 first cancers among male T2D patients (18.7 per 1,000 py) and 605 among females (12.7 per 1,000 py). The risk of any incident cancer in T2D was raised (SIR = 1.14; 95% confidence interval [1.10 - 1.21]), in particular for cancer of the liver (SIR = 1.94 [1.15 - 2.94]) and pancreas (SIR = 1.45 [1.07-1.92]). SIRs decreased markedly with time after T2D diagnosis. In Cox models, adjusting for diabetes duration, body mass index and sex, insulin therapy was related to higher cancer risk (HR = 1.25 [1.17 - 1.33]). No effect was seen for metformin.

**Discussion:**

Our study demonstrates feasibility of record linkage between DMP and cancer registries. These first cohort results confirm previous reports. It is envisaged to enhance this cohort by inclusion of further regions of the state, expansion of the follow-up times, and collection of a more detailed medication history.

## Background

Evidence from epidemiological observation studies suggests that pathophysiological conditions involving hyperinsulinaemia, such as obesity or sedentary behavior, are risk factors for the development of malignant neoplasias [1-4]. Likewise, for type 2 diabetes (T2D), a disorder in which obesity is a major risk factor and insulin resistance an inherent characteristic, recent findings have been accumulated that link this disease and its treatment with the risk of cancer [2,4-11]. In June 2010, an expert consensus report on Diabetes and Cancer was published that assesses the scientific evidence regarding this issue. This report identified numerous unanswered questions in four broad areas. First, more research is required into the specific, especially the less common, cancer types and the impact on cancer prognosis and mortality in T2D including the role of diabetes duration and multidrug therapy. Second, the complex interplay of life style and genetic factors, and their relation over time, needs to be better elucidated. Third, diabetes and cancer may share common predisposing factors, such as hyperglycaemia, hyperinsulinaemia, and inflammation, without being causally related - resulting in the question whether metabolic insulin resistance is accompanied by growth promoting effects of hyperinsulinemia. Last, how can one assess the independent associations between a specific medication and cancer risk, relative to no medication, when the progressive nature of T2D requires adaptation of therapy over time? The expert panel concluded that randomised controlled clinical trials will be unlikely to fully address all these questions, and that multiple well conducted and appropriately designed prospective studies are needed [1,5].

We report here the baseline results from a large cohort study, the 'Diabetes II-to-Cancer' (D2C) cohort, that aims to provide detailed information on the occurrence of incident cancer of various types. It involves T2D patients from a state-wide disease management program who were linked with records from a population-based state cancer registry.

## Methods

### Disease Management Program Diabetes Type 2

The German Statutory Health Insurance (SHI) system consists of more than one hundred sickness funds, which are non-profit insurance companies covering inpatient and ambulatory care as well as pharmaceuticals, and insure about 90 percent of the nation's population. Germany is the only country that has implemented a nationwide primary care-based and physician-sustained disease management program, currently accessible to around 90 percent of the population [12]. Disease management programs (DMP) were introduced in 2003 for patients previously diagnosed with type 2 diabetes. Physician's and patient's participation in the program is voluntary. Primary care physicians enrol patients, and they educate and advise those patients with respect to the management of their disease and use of the health care system. The program includes regular physician-patient consultations at three-month intervals, including a diabetes-specific physical examination, lab tests, patient education, discussion of patient-specific treatment goals, specialist referral if required, documentation of all medical findings in a standardised documentation routine, and treatment according to evidence-based guidelines.

### Epidemiological Cancer Registry NRW

The Cancer Registry of the state of North-Rhine-Westphalia (EKR NRW) collects, links, stores and analyses data about state-wide incident cancer disease. It provides a database for reports and research about the frequency, distribution, and occurrence of cancer diseases in the population of NRW including also survival analyses. It requires, and accepts, with exception only electronic notifications of incident cancer cases. The notification of the first diagnosis of a cancer case by the treating and/or diagnosing physicians is required by law and mandatory. Mortality and survival of cancer patients is assessed on an annual basis by linking cancer cases in the EKR NRW with electronic reports on all deceased individuals in NRW obtained from population registration offices.

For reasons of data confidentiality, the law stipulates that personal identifiers of each cancer case may not be stored as plain text in the registry but only in an encrypted manner. Therefore, encryption procedures were devised for the encryption of each notification of a cancer case as well as for death certificates. Specifically, the personal identifying variables (i.e., family name, first names, birth name, street and house number, and day of birth) are submitted to an initial (one-way) encryption in the notifying office (i.e., hospital, physician's office or pathologist) before the data are released. These initially encrypted data are transmitted over a secure data line to a dedicated server which is located within the special security network of the Cooperation of SHI-based physicians. Here, a second (symmetrical) encryption procedure is completed before the data are forwarded to the EKR NRW. All medical and epidemiological data and part of the personal identifiers (sex, month and year of birth, postal code and place of residence, nationality) remain in plain text and are sent directly via a separate safe network to the EKR NRW. The two parts of each notification are linked again in the registry before entering the internal processing (coding, linkage and best-of-tumour generation). The procedure is labelled 'pseudonymisation' and generates a unique string of characters which allows the unequivocal assignment of specific pseudonyms to the original name [13]. These pseudonyms are the basis for all subsequent record linkage procedures within the EKR NRW. All record linkage procedures are semi-automatic and entirely probabilistic. The estimated completeness of cancer registration in the EKR NRW is over 90% [14].

### Record linkage between DMP data base and EKR NRW

In addition to administrative data, including day of DMP enrolment, the standardised documentation comprised information on anthropometry and physical examination including laboratory tests, type of diabetes medication, duration, symptoms and complications of diabetes, concurrent morbidity, and medical history. Most data of the DMP patients are stored in a central data bank. After details on sex and place of residence, which were kept separately in the SHI files, were linked to the central data bank by individual insurance numbers, records could be submitted to the pseudonymisation process required for the stochastic record linkage with the EKR NRW [13].

### Feasibility study

We report here the results of the first feasibility study that was conducted to enumerate the D2C cohort and establish the procedures of record linkage. We obtained the data of 125,211 DMP patients from one of the major SHI funds, the AOK Nord-West, covering exclusively patients from Westphalia-Lippe, the Northwestern region of NRW. The date of patients' enrolment ranged from June 2003 to the end of July 2008. The feasibility tests were performed on a subpopulation of 31,203 DMP patients residing in the Regierungsbezirk (Administrative District) Münster, because cancer registration in this region was over 95% complete and it was already in full operation when the DMP started in 2003. Cancer registration for the rest of Westphalia-Lippe started in mid 2005 such that other regions reached comparable levels of completeness only from 2007 onwards. We further defined an age range from 40 to 79 years, such that ultimately 27,450 T2D patients were eligible for a record linkage with the cancer registry.

**Figure 1:**
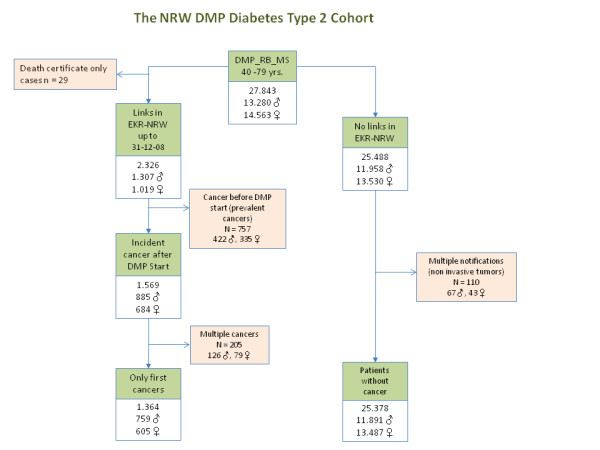
**Selection of study participants for this study**.

The record linkage was run at the end of November 2009. All cohort observation data were censored at the end of 2008 to account for reporting lags. Thus, the time under risk for each individual cohort patient lasted either from day of enrolment until December 31, 2008, or - in the case of cancer occurrence - until the day of cancer diagnosis. There were 27,843 records produced as a result of the linkage procedure (Figure 1). Only cases of a first cancer entered the analysis, multiple cancers were ignored. Diabetics for whom a diagnosis of cancer had been recorded in the EKR NRW before the day of DMP enrolment (prevalent cancers) were excluded; likewise, cancer cases detected exclusively by death certificate (DCO cases) were also excluded. Ultimately, 26,742 patients were available for study analyses. Figure 1 provides a flow chart depicting the selection process of our study participants.

### Statistical Analyses

Cancer cases were counted as incident when a first diagnosis of cancer in the EKR NRW was linked to a DMP patient, and the diagnosis occurred between program enrolment and December 31, 2008. Person-years were used to calculate the time at risk for each individual in the cohort, either as time to incident cancer or time to censoring. In this feasibility study, we had no access (yet) to information on whether a cohort member had died of cancer or any other cause, or had moved out of the area.

For external comparisons, we calculated standardised incidence ratios (SIR) to compare the observed numbers of all cancers, and of specific cancer types, with the expected numbers of cancer cases [15]. The latter were derived from the respective annual cancer incidence rates observed during the observation period in the source population by the EKR NRW. As source population we defined the general population of Regierungsbezirk Münster in the age range 40 to 79 years. Computations were performed using the program proposed by Wood et al. [16] applying five year age groups, combining person-years accumulated with the age-group specific cancer incidence rates in the general population, which was averaged over the calendar period 2003-2007. We computed confidence intervals to reflect the precision of the SIR estimates and selected 99% confidence levels to account for the multiple statistical tests performed on the various cancer entities.

For within-cohort comparisons, we used Cox proportional hazard models to estimate hazard ratios (HR), employing age as the underlying time-scale and age at enrolment as delayed entry point, while adjusting for sex, BMI, diabetes duration as a time-dependent variable, and medication at study entry. The Cox PH models were specified a priori so that 95%-confidence intervals were considered appropriate for their presentation. All analyses were performed with the statistical software SAS 9.2.

## Results

### Baseline characteristics of study participants

The cohort subsample selected for the feasibility assessment consisted of 12 650 men and 14 092 women with an average age of about 63 years in men and 65 years in women. Expectedly for this group, the body mass index was high and about one in five men and one in ten women were active smokers. The median time under observation was approximately 3.5 years. One in four patients received no anti-diabetic medication at the time of enrolment in to the DMP (Table 1).

**Table 1:** Baseline description of the D2C cohort

	Men		Women	
N	12 650	47,3%	14 092	52,7%
Median follow-up time (years)	3.54	[0.3 - 5.8]	3.79	[0.2 - 5.8]
Mean Age (years)	62.6	[40-79]	65.2	[40-79]
Median BMI (kg/m^2^)	29.7	[16.3 - 44.9]	31.0	[16.2 - 44.9]
Current smokers	2934	23.2%	1659	11.8%
Year of enrolment in DMP				
2003/2004	5 845	46.2%	6 838	48.5%
2005/2006	3 148	24.9%	3 407	24.2%
2007/2008	3 657	28.9%	3 847	27.3%
Anti-diabetic medication				
No medication	3 041	24.0%	3 465	24.6%
Metformin only	4 580	36.2%	5 064	35.9%
Any other oral antidiabetic drug	1 782	14.1%	1 868	13.3%
Human insulin (alone or with metformin)	2 558	20.2%	3 018	21.4%
Insulin Analogues (alone or with insulin and/or metformin)	690	5.5%	677	4.8%

### Crude cancer incidence rates

We observed 1364 cases of incident cancer during a total follow-up time of 88 773 person-years. The crude cancer incidence rate was higher in men (18.4 per 1000 person-years) than in women (12.7 per 1000). Numbers and rates for specific cancers are given in Table 2.

**Table 2:** Numbers of first invasive cancers and crude cancer incidence rates in the T2D cohort

Cancer type (ICD-10)	Men	Women
	N = 12.650; 41.170 py	N = 14.092; 47.603 py

Any cancer (excluding C 44*)	759 (18.4 per 1 000 py)	605 (12.7 per 1 000 py)
Liver (C 22)	23 (0.6 per 1 000 py)	10 (0.2 per 1 000 py)
Pancreas (C 25)	22 (0.5 per 1 000 py)	27 (0.7 per 1 000 py)
Breast (C 50)	2 (0.05 per 1 000 py)	129 (2.7 per 1 000 py)
Prostate (C 61)	132 (3.2 per 1 000 py)	-
Colorectal (C18 - C21)	107 (2.6 per 1 000 py)	88 (1.8 per 1 000 py)
Lung (C34)	121 (2.9 per 1 000 py)	42 (0.9 per 1 000 py)

*C44 (Other malignant neoplasms of skin)

### External comparisons with general population

The standardised incidence ratios (SIR) revealed that the rate of cancer occurrence was higher in this cohort of diabetics than in the general population as reflected by an SIR = 1.14 (99% confidence interval [1.04-1.21]; Table 3). The SIR was specifically raised for cancers of the liver (SIR = 1.94) and for pancreas cancer (SIR = 1.45). Conversely, the risk of prostate cancer was clearly lowered among diabetics (SIR = 0.65). Incident cancers of the breast, colorectum and lung were not raised. Results were largely consistent for men and women.

**Table 3:** Standardised incidence ratios (SIR) for invasive cancers in the D2C cohort

Cancer type (ICD-10)	SIR					
	
	Men	99% CI	Women	99% CI	All	99% CI
Any cancer (excluding C 44*)	1.11	[1.01-1.21]	1.18	[1.07-1.31]	1.14	[1.04-1.21]
Liver (C 22)	1.88	[1.02-3.15]	2.08	[0.78-4.46]	1.94	[1.18-2.99]
Pancreas (C 25)	1.27	[0.68-2.15]	1.63	[0.93-2.63]	1.45	[0.97-2-06]
Breast (C 50)	-	-	0.86	[0.68-1.07]	-	-
Prostate (C 61)	0.65	[0.52-0.82]	-	-	-	-
Colorectal (C18 - C21)	1.00	[0.77-1.23]	0,97	[0.73-1.28]	0.99	[0.81-1.19]
Lung (C34)	1.04	[0.81-1.31]	1.06	[0.69-1.56]	1.05	[0.85-1.27]

*C44 (Other malignant neoplasms of skin)

With regard to cofactors, age did not modify the risk of cancer among diabetics while body mass index seemed to have a very moderately increasing impact (Table 4). Of note, however, the duration of diabetes was strongly and inversely associated with the occurrence of any invasive cancer: this risk was markedly higher within the first year after diabetes had been diagnosed.

**Table 4:** Standardised incidence ratios (SIR) for all invasive cancers, by age, BMI and diabetes duration

Variable	SIR	99% CI
Age		
< 60 years	1.14	[0.94-1.38]
≥ 60 years	1.14	[1.06-1.23]
T2D duration at study entry		
< 1 year	1.27	[1.08-1.47]
1 - 3 years	1.13	[0.94-1.34]
> 3 years	1.10	[1.00-1.21]

### Internal comparisons within cohort of diabetics

We analysed further how patient characteristics influenced the risk of cancer within this cohort of diabetics. The risk of cancer was lower among diabetic women. Obesity seemed inversely related to cancer risk among diabetics; similarly, diabetes duration was also inversely associated with occurrence of any cancer. Of the anti-diabetic medication taken by T2D patients, only insulin, either alone or in combination with metformin, seemed to raise the cancer risk significantly (HR = 1.25, 95% confidence interval [1.17 - 1.33]; Table 5).

**Table 5:** Patient characteristics with impact on total cancer incidence

Variable	HR	95%-CI
Female sex	0.69	[0.65-0.72]
BMI (≥ 30 kg/m^2^)	0.90	[0.86-0.95]
Diabetes duration more than 2 years	0.79	[0.73-0.86]
Metformin (only)	0.95	[0.90-1.01]
Insulin (including combination with metformin)	1.25	[1.17-1.33]
Analogues (including combination with insulin/metformin)	0.89	[0.79-1.01]

## Discussion

Our study had two aspects: one was to test the feasibility of a pseudonymised procedure for the linkage of records from different data collection systems (DMP T2D and EKR NRW), and the second was to explore the incidence of cancer in this cohort over a fairly short time of observation. The specific record linkage procedures had been positively evaluated before comparing pseudonymised with plain text personal identifiers which had been obtained from standardised and structured cancer case notifications in the EKR NRW. Our study shows that other sets of data, collected for purposes other than cancer registration, can also be effectively linked by using these procedures. Specific prerequisites and technical details of this procedure will be described somewhere else (in preparation). In general, the procedures worked smoothly and efficiently.

Lending support to the validity and credibility of our study, these descriptive and preliminary results with regard to cancer incidence among T2D patients appear to essentially confirm previous reports. Thus, the incidence rate for any type of cancer was found to be raised among diabetics, when compared with the source population, and the size of the elevation was similar to risk ratios reported in recent meta-analyses and the 2010 consensus report [1,4,5,17]. Similarly, the elevations of risk for cancers of the liver and pancreas, and the decreased risk for prostate cancer have also been consistently observed in T2D before [4,17]. We noted, however, that the SIR was particularly high in the first year after diabetes was diagnosed. This may be attributable on one hand to a detection bias which arises when freshly diagnosed diabetics increase the frequency of their contacts with the health system or when they are initially submitted to more intensified medical examinations: In such a situation, prevalent cancers are more likely to be detected. On the other hand, reverse causality, that is, cancers compromising glucose-metabolism, may offer another plausible explanation of such findings. Of note, this temporal pattern is rather common and has been discussed recently [2,7,18].

Investigating the factors that impact on cancer occurrence within the cohort of diabetic patients, we found - apart from diabetes duration which had the strongest inverse influence - that insulin therapy, either as a monotherapy or in combination with metformin, increased the cancer risk by about 25%. This finding is in line with previous reports [4,6,7,11,18]. We were unable to identify raised cancer risk among cohort members who took insulin analogues, either alone or in combination. These drugs, specifically the long acting modality glargine in higher dosages, have been recently suspected of being related to raised cancer risks [2,6,7,11,19]. Unfortunately, the number of T2D patients taking these medications was low, the distinction between long and fast acting analogues was presently not possible, nor did we - as yet - have data on drug dosages. Furthermore, and in contrast to several recent reports [1,2,5], we could presently not identify a significant inverse association between metformin use and cancer incidence.

Our study has several limitations. The follow-up period was rather short for the effects of therapy and cancer therapy to fully develop missing cases with later onset. Likewise, taking into account the reporting lag inherent in any system of cancer registration, population-based completeness of notification can be expected about 18 months after cancer diagnosis; therefore, a certain degree of underestimation of cancer occurrence in this cohort is likely. Another bias has probably arisen from the, as yet, unavailability of data on the mobility and mortality of this cohort. T2D patients are likely to have a higher rate of competing risks and mortality, e.g. due to cardiovascular diseases, than the general population: this would result in lower SIR in diabetics, and it will probably also tend to lower the relative risk estimates within the cohort. On the other hand, less seriously ill diabetics may have moved out of the catchment area of the register and were thus lost to follow-up; this might have resulted in spuriously increased hazard ratios. However, the migration rates in this age group in NRW are generally low and we do not perceive this mobility to be largely differential; therefore, the effect on SIR and HR is expected to be small. In summary, the directions of these potential biases tend mostly to reduce the observed ratios, and thus our estimates are probably presumed to conservative rather than overemphasizing.

The D2C cohort will be continued. The number of patients will be increased by patients residing in other areas of Westphalia-Lippe as cancer registration became complete in these areas from 2007 onwards. This will be complemented by repeating follow-ups annually to increase the total person-time. Finally, we are presently attempting to develop a system that will enhance the availability of data on drug use incorporating type, dosage and duration for each individual cohort member. In addition, means are being explored to implement a pseudonymised record linkage of death records, routinely provided to the EKR NRW, with the DMP cohort for the assessment of T2D patients' mortality.

## Conclusions

Our study demonstrates feasibility of record linkage between DMP and cancer registries. The first cohort results confirm previous reports on the association of T2D with cancer and the role of insulin therapy. It is envisaged to enhance the informativeness of this cohort by inclusion of further regions of the state, expansion of the follow-up times, and collection of a more detailed medication history.

## Competing interests

The authors declare that they have no competing interests.

## Authors' contributions

HWH planned the study, supervised the analyses and wrote a first draft of the manuscript. HK and WUB contributed to data analysis and contributed to the manuscript. JW contributed to the statistical methods and analyses, and commented on previous versions of the manuscript. All authors read and approved the final manuscript.
